# Metabolomics and Transcriptome Analysis of Rapeseed Under Salt Stress at Germination Stage

**DOI:** 10.3390/cimb47070481

**Published:** 2025-06-24

**Authors:** Menglin Zhou, Xi Song, Qingqing Yu, Bingbing Dai, Wei Zhou, Xiaofei Zan, Wuming Deng

**Affiliations:** Nanchong Academy of Agricultural Sciences, Nanchong 637000, China

**Keywords:** rapeseed, salt stress, metabolomics, transcriptome

## Abstract

Salt stress is a significant abiotic factor that adversely impacts the yield of rapeseed (*Brassica napus* L.). Under salt stress conditions, the growth of rapeseed is markedly inhibited. This study integrates transcriptomic and metabolomic analyses to elucidate the molecular and physiological mechanisms underlying the salt stress response during the germination of the rapeseed variety ZS11. Metabolomic analysis revealed 175 differentially expressed metabolites, predominantly comprising amino acids, carbohydrates, and organic acids. Transcriptomic analysis highlighted the crucial roles of plant hormones and phenylpropanoid biosynthesis in enhancing the salt stress resistance of rapeseed. Comprehensive multi-omics analysis identified phenylpropanoid metabolism (*p* < 0.001), amino acid metabolism (FDR < 0.01), and carbohydrate metabolism (|log_2_FC| ≥ 2) as the most significantly affected pathways. Crucially, we demonstrate that early-stage phenylpropanoid activation in hypocotyls dominates salt adaptation during germination. These findings provide actionable targets for molecular breeding and novel insights for optimizing crop establishment in salinized agroecosystems.

## 1. Introduction

As one of the four major oil crops globally, rapeseed serves not only as a significant source of vegetable oil but also as an important source of feed protein. Currently, China relies heavily on imports for its edible oil supply, which poses considerable threats and challenges to food safety. Expanding the planting area of rapeseed presents an effective short-term solution to address this issue. In China, the total area of saline-alkali land amounts to approximately 99.13 million hectares, with around 36.667 million hectares experiencing severe salinization. These affected regions are predominantly located in Northeast China, North China, and Northwest China, as well as the upper and middle reaches of the Yangtze River and the Binhai New Area [1].

Salt stress, often referred to as salt damage, can trigger excessive production of reactive oxygen species (ROS), ultimately leading to oxidative damage of organelles and membrane components, and in severe cases, resulting in cell and plant death. The antioxidant defense system serves to protect plants from salt-induced oxidative damage by detoxifying ROS and maintaining a balance in ROS production under salt stress conditions [2]. When the salt concentration in the soil reaches 0.2–0.5%, it poses a significant threat to plant growth, development, and yield [3,4]. Globally, many irrigated lands face the dual challenges of soil salinization and waterlogging, with over 20% of the total global irrigation area currently affected by these issues. If left unaddressed, projections indicate that more than 50% of the world’s arable land may experience salinization by 2050 [5]. Due to high costs, the cultivation or screening of salt-tolerant crops is considered the most economical, feasible, and effective approach to improve and utilize saline-alkali land resources, thereby ensuring the long-term sustainability of these soils.

Germination represents a critical initial phase in the plant life cycle, with its significance manifested in three key aspects. Physiological foundation establishment: Seed imbibition activates enzymatic systems and initiates hydrolysis of reserve substances (starch and proteins), providing the energy and nutrients for the radicle and plumule development. Stress resilience priming: This hypersensitive stage to environmental stresses (e.g., salinity and drought) shapes subsequent adaptive potential through early activation of phenylpropanoid metabolism and antioxidant systems (SOD/CAT) [6,7]. Agronomic performance determinant: The germination rate directly impacts field emergence efficiency and production costs. The active metabolic reprogramming during this phase makes it the optimal entry point for elucidating stress adaptation mechanisms.

The salt tolerance of rapeseed involves complex molecular mechanisms. Although numerous genes related to salt stress have been identified, the genetic and molecular mechanisms underlying its response to salt stress remain unclear. Notably, many previously unreported genes have been found to play a role in the salt stress response. This study aims to elucidate the mechanisms of salt stress adaptation in B. napus and provide valuable references for the genetic improvement of the salt tolerance in this species.

## 2. Materials and Methods

### 2.1. Materials, Treatment Methods, and Sample Preparation

Zhongshuang 11 (ZS11), an exceptional inbred line of Brassica napus, served as the experimental material. The seeds were obtained from the Institute of Oil Crops, Chinese Academy of Agricultural Sciences. The germination experiment was conducted in a rapeseed tissue culture room. The day and night temperatures were maintained at 25 ± 1 °C, with a light/dark cycle of 12/12 h, a light intensity of 7000 Lux, and humidity levels ranging from 60% to 80%. Randomly selected culture dishes with a diameter of 10 cm were washed with tap water and then washed with pure water. After drying, 8 layers of absorbent paper were placed at the bottom of the culture dish. In order to germinate, 12 mL of deionized water was added to each culture dish and randomly placed in different locations in the growth chamber. After 7 days, seedlings of uniform size were randomly selected from the treatment group in the petri dish and transplanted into 12 mL of 200 mM NaCl solution for salt stress treatment. The control group was similarly transplanted into an equal volume of deionized pure water to continue culturing. A total of 100 mature, plump, and uniformly sized seeds were placed in each dish, arranged neatly, and then transferred to the tissue culture room for germination. The petri dishes were sealed with Parafilm to prevent water evaporation, and three repetitions were established.

On the third day of the post-treatment, three to five seedlings exhibiting relatively consistent growth—encompassing both aboveground and underground parts—were selected from both the treatment and control groups and combined into biological samples. Three biological samples were prepared for transcriptomic and metabolomic analyses, with the samples being snap-frozen in liquid nitrogen and subsequently stored at −80 °C for future use. The samples were measured and analyzed by Proneis Biotechnology. The control group was categorized into CK_1, CK_2, and CK_3, while the treatment group was designated as T1_1, T1_2, and T1_3.

Proline concentration was determined using the ninhydrin method, with measurements taken at 520 nm using an ultraviolet spectrophotometer. Catalase (CAT) activity was calculated as the amount of H_2_O_2_ decomposed, expressed as mgH_2_O_2_ g^−1^ protein min^−1^.

### 2.2. Metabolomic Analysis of Rapeseed Under Salt Stress

The LC-MS analysis platform was employed to investigate the metabolomics of the samples. Initially, the samples underwent pretreatment to eliminate proteins and impurities, facilitating the extraction of metabolites. These metabolites were subsequently detected using LC-MS in both positive and negative modes, yielding MS and MS/MS data. Progenesis QI software (v2.3; Waters Corporation, Milford, CT, USA) was utilized for metabolite annotation and data preprocessing, incorporating in-house spectral libraries and HMDB database matching. Ultimately, a metabolite list and data matrix were generated, from which differential metabolites were identified using the two-tailed Student’s *t*-test with Benjamini–Hochberg false discovery rate correction and VIP criteria (OPLS-DA model validation by 7-fold cross-validation; VIP > 1, *p* < 0.05 adjusted). Furthermore, advanced analyses, including pathway enrichment (hypergeometric test with FDR correction), association analysis (Spearman’s rank correlation), and weighted gene co-expression network analysis, were conducted to explore the biological significance of the differential metabolites [8].

### 2.3. Transcriptome Analysis of Rapeseed Under Salt Stress

After the total RNA was extracted from the tissue samples (RNA integrity number ≥ 8.0, Agilent 2100 Bioanalyzer, Agilent Technologies, Santa Clara, CA, USA), the mRNA was enriched using Oligo dT beads. The mRNA was then fragmented (~300 bp fragments), which was followed by cDNA synthesis via reverse transcription (SuperScript^TM^ IV, Thermo Fisher Scientific, Waltham, MA, USA) with adaptor ligation. Library preparation was performed using the NEBNext^®^ Ultra^TM^ II RNA Library Prep Kit (Illumina, San Diego, CA, USA) with 150 bp paired-end sequencing on NovaSeq 6000 (Illumina, San Diego, CA, USA), yielding ≥50 million reads per sample (Q30 > 90%). The experiment included three biological replicates. Following the sequencing, the raw reads were processed with FastQC v0.12.1 for quality control and Trim Galore! v0.6.7 (adapter removal, Phred score ≥ 30). Clean reads were aligned to the rapeseed ZS11 reference genome (HISAT2 v2.2.1 with default parameters) and quantified using featureCounts v2.0.3. Differentially expressed genes (DEGs) were identified with DESeq2 v1.38.3 using thresholds of |log2 fold change| >1 (corresponding to 2-fold expression difference) and Benjamini–Hochberg false discovery rate (FDR) < 0.05. All transcripts were annotated against six databases (NR, Swiss-Prot, Pfam, COG, GO, KEGG; DIAMOND v2.1.6 with e-value < 1 × 10^−5^) and statistically analyzed (clusterProfiler v4.8 for enrichment). In the process of differential expression gene detection, |log_2_FC| ≥ 2 and FDR < 0.05 were used as the screening criteria [9,10,11,12].

### 2.4. Correlation Analysis of Transcriptome and Metabolome

The metabolic pathway information of the entire biological system can be visualized using iPath2.0 (http://pathways.embl.de, accessed on 25 September 2024) [13]. To further examine the changes and correlations between the metabolites and genes, we identified and visualized the top 10 KEGG pathways with the highest number of genes (log_2_FC > 1, *p* < 0.05, FPKM > 10) and metabolites (VIP > 1, log_2_FC > 1, *p* < 0.05) from this experiment. The differentially expressed genes obtained from the transcriptomics and the differentially abundant metabolites from the metabolomics were integrated into a KEGG pathway diagram using Cytoscape (v3.9.1), al-lowing for an intuitive and comprehensive representation of the pathway data [14,15].

## 3. Results

### 3.1. Effects of Salt Stress Development of Rapeseed

**Figure 1:**
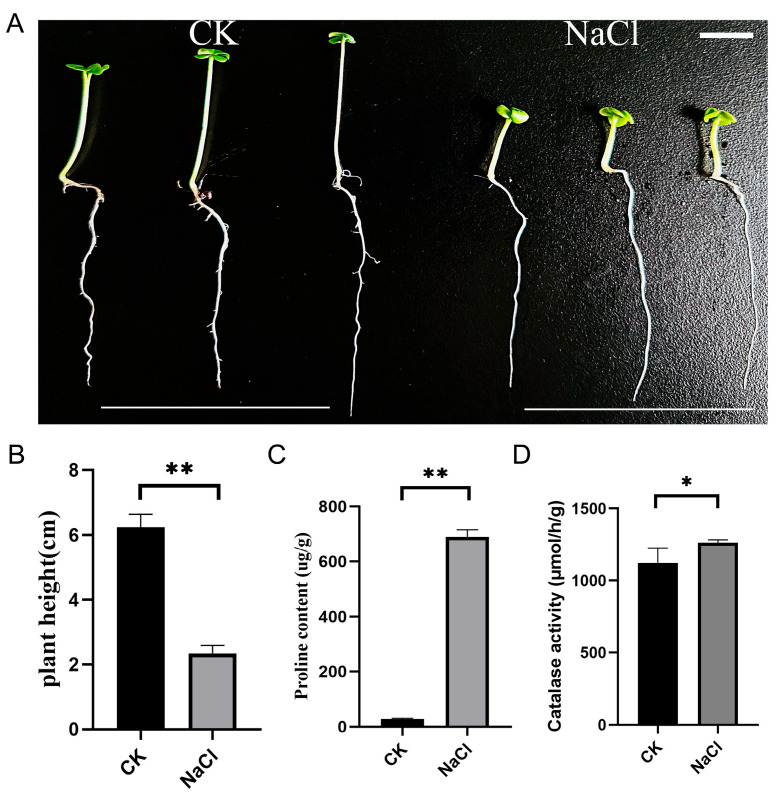
The effect of NaCl treatment on the growth of the ZS11 seedling. (**A**) Seedling phenotype. (**B**) Plant height. (**C**) Proline content. (**D**) Catalase activity. Analysis of variance and Tukey test were performed. The significance levels were expressed as * and **, corresponding to *p* < 0.05 and *p* < 0.01, respectively. Scale: 2 cm.

To investigate the effect of salt stress on the early development of rapeseed seed-lings, we utilized ZS11 seeds germinated in petri dishes. Previous studies have demonstrated that ZS11 can germinate normally at low concentrations of NaCl (100 mM); however, ZS11 seeds fail to germinate at a concentration of 200 mM [16]. Consequently, our treatment protocol involved germinating the seeds in distilled water for 7 days, followed by exposure to 200 mM sodium chloride for 3 days. The results indicated that after the treatment with 200 mM NaCl, the seedlings in the treatment group exhibited impaired growth compared to the control hypocotyls (Figure 1A,B). Salt stress resulted in a significant increase in the proline content and catalase activity in the seedlings (Figure 1C,D).

### 3.2. Study on Metabolic Changes of Rape Under Salt Stress

**Figure 2:**
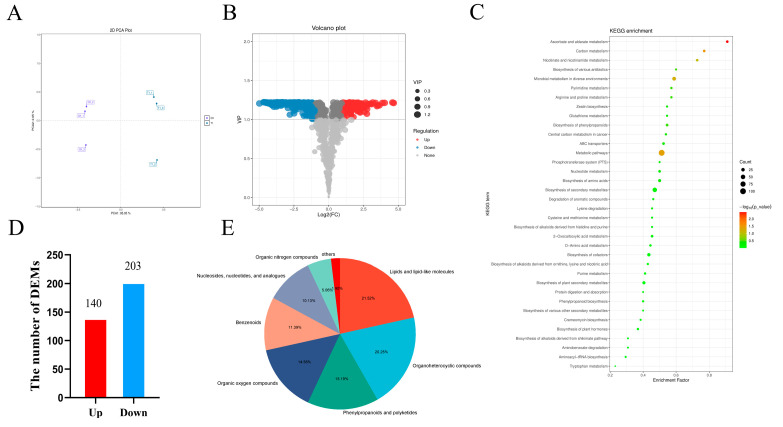
The effect of the NaCl treatment on the rapeseed metabolism was assessed through several analytical approaches. (**A**) PCA was conducted to compare the metabolites in the control samples versus those treated with NaCl. (**B**) A volcano plot was generated to illustrate the DEMs. (**C**) A KEGG enrichment plot was created to analyze the pathways associated with the differential metabolites. (**D**) The number of DEMs. (**E**) The classification of the DEMs was performed to categorize their variations.

To fully understand the response of the rapeseed to the salt stress during germination, we conducted an extensive targeted metabolomics analysis utilizing LC-MS. Principal component analysis (PCA) was performed on the metabolomic spectrum to provide insights into different groups (Figure 2A). The first and second principal components accounted for 95.05% and 2.05% of the inter-sample variance, respectively (Figure 2A). The partial least squares discriminant analysis (PLS-DA) score map (Figure S1) effectively distinguishes the metabolic spectrum under salt treatment. DEMs were screened using P1, FC > 1, or FC < −1 as screening criteria. A total of 343 metabolites were detected and presented in the volcanogram (Figure 2B). Compared to the CK, 140 metabolites were up-regulated, while 203 were down-regulated in the treatment group (Figure 2D and Table S1).

To further investigate the biological processes in response to salt stress, we focused on core DEMs (Table S2) exhibiting log_2_ fold changes (log_2_FC) greater than 2 or less than −2. We analyzed a selected set of 175 DEMs along with their annotation results, categorizing them into several groups (Figure 2E). The first group includes amino acids, such as ornithine, cysteine, aspartic acid, threonine, and glutamic acid. Notably, while the amino acids like ornithine (log_2_FC = 21) and cysteine (log_2_FC = 20) were induced by salt stress, the levels of the other amino acids were significantly down-regulated. This observation suggests that salt stress inhibits the normal metabolic processes of amino acids in rapeseed. The second group comprises sugars and alcohols, including glucose, alditol, xylose, isomaltulose, and lactobiose. The majority of carbohydrates were found to be down-regulated, indicating that salt stress hinders the formation of sugars and alcohol. The third group consists of organic acids, such as methyl-glutaric acid, citric acid, malic acid, quinic acid, and fumaric acid. The levels of citric acid (log_2_FC = −4), fumaric acid (log_2_FC = −3), and malic acid (log_2_FC = −4) in the TCA cycle decreased significantly under salt stress, suggesting that the energy metabolism pathway in rapeseed is adversely affected by this stress. Collectively, these results indicate that salt stress primarily regulates the metabolism of specific amino acids, carbohydrates, and organic acids (Figure 2C).

### 3.3. Transcriptome Identification of Salt Stress Response Genes

**Figure 3:**
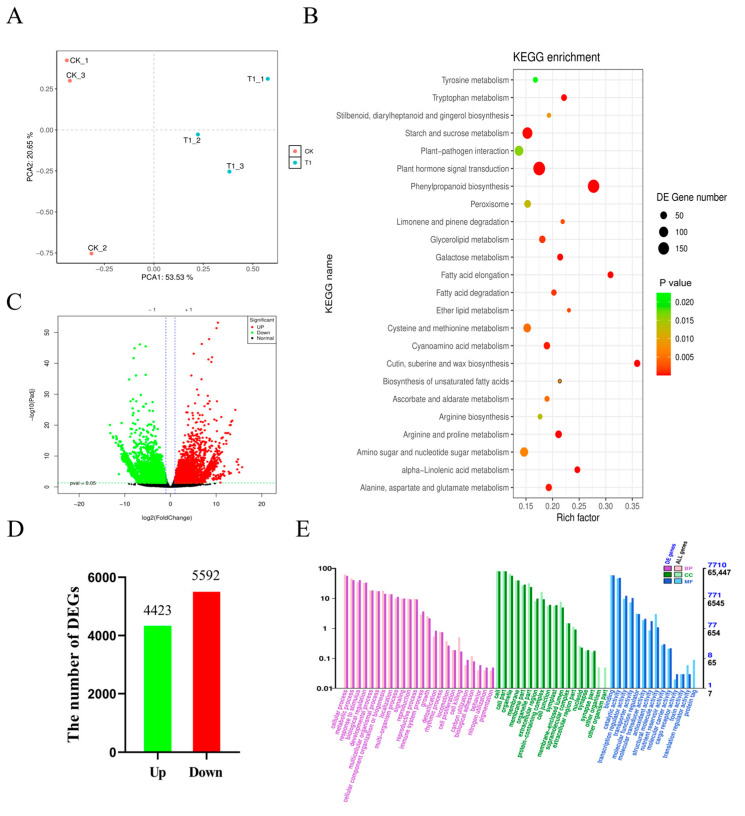
The effect of the NaCl treatment on the rapeseed transcriptome is illustrated in several analyses. (**A**) A principal component analysis (PCA) was conducted to compare the control and NaCl-treated samples. (**B**) A scatter plot depicts the KEGG pathway enrichment of differentially expressed genes. (**C**) A volcano plot illustrates the differential expression of genes. (**D**) The number of DEGs. (**E**) The GO annotation classification statistics for the differentially expressed genes are presented.

To identify the genes that respond to salt stress, RNA-seq analysis was conducted on rapeseed. A *p*-value threshold of 0.05 was established to determine the significance of the gene expression differences between the samples. A total of 10,015 differentially expressed genes were identified between the CK and T1 treatments, comprising 4423 up-regulated and 5592 down-regulated genes (Figure 3D). Notably, the number of down-regulated genes exceeded that of the up-regulated genes (Table S3 and Figure 3C). PCA of the transcriptome data indicated that PC1 effectively separated the T1 treatment from the CK group, with PC1 accounting for 53.53% of the variation and PC2 accounting for 20.65% (Figure 3A). These results suggest that a significant portion of the transcriptome undergoes changes in response to salt stress.

The Gene Ontology (GO) method was employed to classify the functions of the DEGs under salt stress. The GO annotation system comprises three primary branches: biological processes (BP), molecular functions (MF), and cellular components (CC) (Figure 3E). According to the KEGG analysis, a total of 3280 DEGs were found to overlap with the KEGG pathway (Table S4). Furthermore, the pathways that contained the highest number of mapped genes included plant hormone signal transduction, phenylpropanoid biosynthesis, starch and sucrose metabolism, amino acid biosynthesis, and carbon metabolism (Figure 3B and Table S5). By integrating the results of pathway functional enrichment analysis, DEGs functional annotation, and expression levels at varying salt concentrations, 44 candidate genes were identified (Table S6).

### 3.4. Joint Analysis of Metabolomics and Transcriptomics

**Figure 4:**
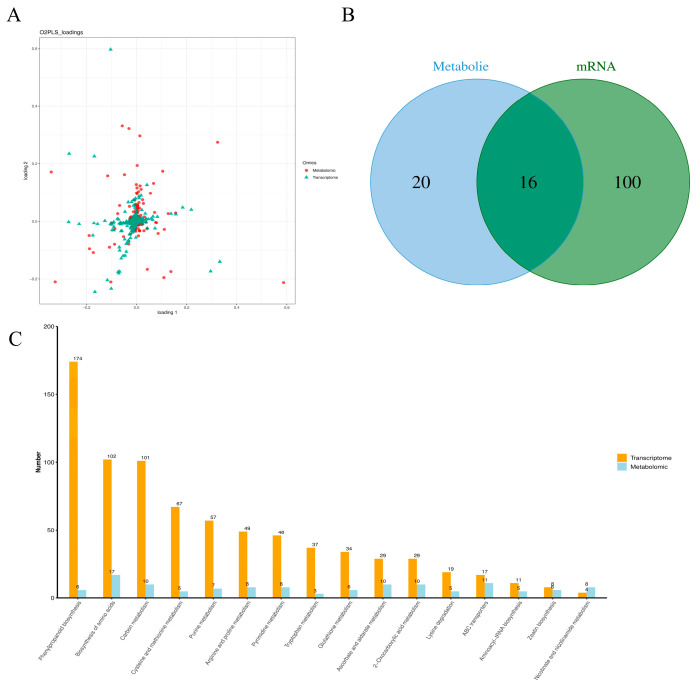
Correlation analysis of the metabolomics and transcriptomics data is presented. (**A**) The O2PLS loading plot illustrates the joint changes observed between the transcript and metabolite data sets. (**B**) A Venn diagram displays the DEGs and DEMs. (**C**) The analysis of transcriptomics and metabolomics has identified the top 10 KEGG pathways that exhibit the highest number of DEGs and DEMs.

The biological process is complex and holistic, and the joint analysis of multi-omics data is more conducive to the study of phenotype and biological process regulation mechanism of biological models. In order to further understand the correlation between the salt stress response metabolome and the transcriptome, we used the loading values of DEMs and DEGs to show the overlap of the two omics. The analysis showed that these two omics were highly correlated (Figure 4A). The Venn diagram shows that the pathways from the DEMs partially overlap those from the DEGs (Figure 4B). The DEMs and DEGs were mainly enriched in ‘phenylpropanoid metabolism’, ‘amino acid metabolism’, and ‘carbon metabolism’(Figure 4C).

**Figure 5:**
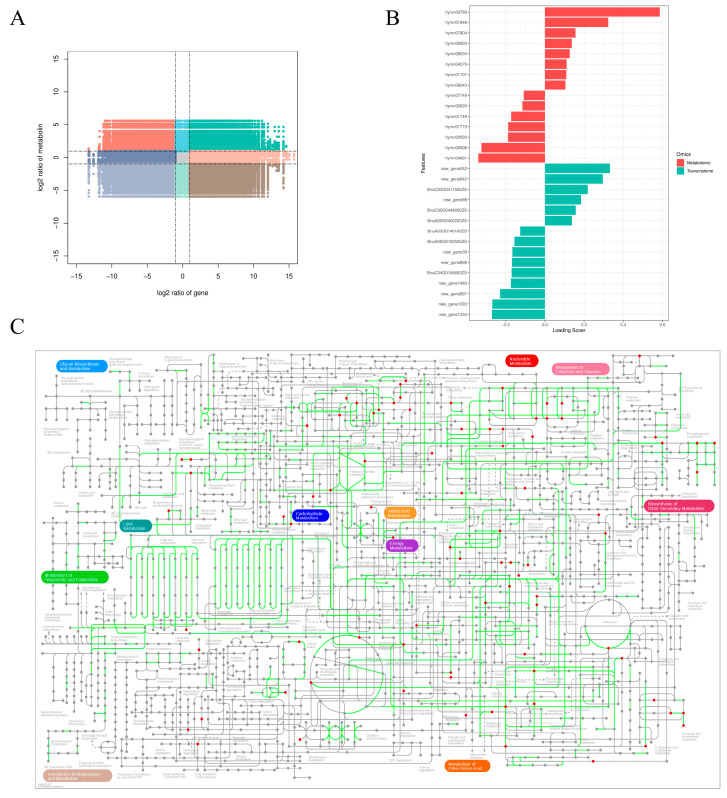
Integration analysis of DEGs and DEMs. (**A**) Correlation Analysis Nine Quadrant Chart. (**B**) The loading value maps of the first 15 genes and metabolites. (**C**) The iPath integrated analysis diagram.

Considering that the abundance of metabolites is determined by the transcript level, we also performed a correlation analysis between the transcriptome and the metabolome. Correlation analysis was performed on the genes and metabolites detected in each differential group, and the Pearson correlation coefficient of genes and metabolites was calculated. The difference multiples of gene metabolites in each differential group were displayed by a nine-quadrant diagram (Figure 5A). Through iPath analysis, DEGs and DEMs were associated with ‘carbohydrate metabolism’, ‘amino acid metabolism’, ‘energy metabolism’, and ‘lipid metabolism’. (Figure 5C and Table S6) is relevant. In order to study the robust changes of metabolites, we conducted a correlation test between the core changed DEMs and the main DEGs. The loading values of the top 15 DEMs and DEGs were plotted (Figure 5B and Table S7). The results show that these DEMs are clustered together and share common DEGs. In summary, correlation analysis showed that these DEGs may play a direct or indirect regulatory role in the metabolism of key DEMs.

To further explore the transcriptional regulation of rapeseed under salt stress, we conducted a detailed study of the differentially expressed genes (DEGs) associated with the phenylpropanoid metabolic pathway (map00940). This finding corroborates the enrichment of DEGs and differentially expressed metabolites (DEMs) in carbohydrate metabolism, identified in our iPath analysis. The accompanying figure illustrates the overall layout of phenylpropanoid metabolism under salt treatment, as well as the relative changes of DEMs (Table S2). Notably, the levels of the intermediate metabolites related to the phenylpropanoid metabolic pathway, including cinnamic acid, p-coumaric acid, and flavonoids, were generally elevated under salt stress. These findings suggest that phenylpropanoid metabolism may be regulated through the enhanced synthesis of cinnamic acid, p-coumaric acid, and flavonoids in response to salt stress. Additionally, we summarized the expression patterns of the DEGs involved in metabolic pathways such as phenylpropanoid metabolism (Table S5). These DEGs exhibit a strong correlation with the aforementioned DEMs.

## 4. Discussion

With the rapid advancements in modern molecular biology, the investigation of plant salt tolerance mechanisms has progressed to encompass transcriptomics, proteomics, metabolomics, and ionomics [17]. “Omics” studies offer a robust methodology for identifying salt-tolerance genes and discovering marker metabolites [18]. Unique genetic resources serve as the foundation for omics research. In this study, the characteristic rapeseed variety ZS11 was selected for transcriptomic and metabolomic analysis. Although ZS11 is recognized as an excellent conventional rapeseed variety, it exhibits significant sensitivity to salt stress [16,19]. Enhancing the salt tolerance of ZS11 could mitigate its limitations and facilitate its cultivation in saline-alkali regions.

In dicotyledonous crops, such as Brassica napus, the germination period is defined as the interval from the onset of the seed germination until the cotyledons flatten, typically lasting 7 to 10 days. This period is crucial for assessing the salt tolerance of the crops for several reasons: (1) It marks the initial stage of crop establishment, which influences the growth quality of individual plants and the yield components during the seedling establishment and subsequent developmental phases, ultimately affecting the overall output [20,21]. (2) The germination period also represents a transition from a relatively static state to one of active metabolism. During this process, high salinity can impede seed water absorption and induce physiological drought through osmotic stress, while also accumulating in germinating seed tissues, leading to ion toxicity. Notably, different genotypes of various crops exhibit significant variability in their resistance to salt stress [20]. (3) Salt damage is distinct from other abiotic stresses, such as humidity and drought damage, which are typically linked to regional environmental changes and inflict harm on crops for a limited duration. In contrast, salt damage arises from the challenges posed by saline-alkali soils, exerting persistent negative effects on the crop root system throughout the entire growth period [22]. Consequently, this study focuses on implementing salt stress treatment during the germination period to investigate the physiological and metabolic changes in rapeseed under such conditions.

Metabolic pathway analysis of Brassica napus (ZS11) seedlings subjected to 200 mM NaCl stress (Figure 3) reveals significant alterations in various metabolic pathways associated with the reduction of multiple metabolites. These include organic acids (such as citric acid, malic acid and its derivatives, butyric acid and its derivatives, and fumaric acid), carbohydrates (including sugars and alcohols), amino acids and their derivatives, lipids, and alkaloids (Table S2). The top 20 metabolites exhibiting the most significant changes in positive ionization mode are predominantly amino acids and their derivatives (including L-ornithine, L-cysteine, L-tyramine, L-aspartic acid-O-diglucoside, and aspartate di-O-glucoside), as well as organic acids (such as D-erythrolactone, muconic acid, and trans-muconic acid) and phenolic acids, sugars, and alcohols. Conversely, the top 20 metabolites showing the most significant changes in negative ionization mode primarily consist of sugars and alcohols (including D-glucophosphonic acid, D(+)-glucose, phosphate, and threose acid salts), amino acids and their derivatives, organic acids, alkaloids, lignans, and phenolic acids (Table S2). The accumulation of amino acids enhances plant stress resistance by promoting the detoxification of reactive oxygen species and regulating pH and osmotic balance [23]. Under alkaline salt stress, the levels of proline, glutamic acid, histidine, and phenylalanine increased. Organic acid metabolism, particularly fatty acid accumulation, along with amino acid metabolism, are crucial metabolic pathways in rape roots under alkaline salt stress [24]. In this study, the content of certain amino acids, such as ornithine and cysteine, increased, while the levels of most other amino acids and their derivatives, including aspartic acid, threonine, glutamic acid, and histidine, decreased significantly. During abiotic stress, low-abundance amino acids are not synthesized; instead, they accumulate and are degraded due to increased protein turnover under conditions that induce carbohydrate starvation, such as salt stress [25]. The results indicate that proline levels significantly increase during seed germination in response to salt stress, and this accumulation of osmotic agents in cells is associated with a reduction in ROS accumulation.

The role of carbohydrates in salt tolerance has been extensively studied. At the physiological level, osmotic regulation serves as an adaptive mechanism for salt tolerance, enabling the maintenance of expansion pressure under stress conditions. In response to salt stress, plant cells synthesize and accumulate various small-molecule organic compounds, such as proline, betaine, sugars, polyols, and polyamines, to maintain intracellular water potential [26]. The increased levels of mannitol, sorbitol, inositol, and their methylated derivatives are significant for improving salt tolerance. Additionally, the application of trehalose and polyols can prevent the oxidation of salt-binding lipids and reduce the accumulation of ROS [27]. Under saline-alkali stress, plants can induce the secretion of a substantial amount of organic acids, which serve a buffering role, enabling plants to resist environmental changes and maintain intracellular pH stability and ion balance [28]. Under saline-alkali stress, tomato roots and leaves maintain ion balance by promoting the synthesis of organic acids, including citric acid, lactic acid, acetic acid, succinic acid, malic acid, and oxalic acid [29]. Organic acids are important intermediates of carbon metabolism in plant cells and play a crucial role in regulating overall plant cell physiology. Most phenolic acids exhibit beneficial nutritional effects and can function as antioxidants, mitigating cell damage caused by free radical oxidation. In plants subjected to salt stress, increases in antioxidant content and phenolic acid concentrations, along with enhanced antioxidant capacity, are frequently observed, suggesting a potential correlation among these variables [30]. This study found significant increases in phenolic acids, including ferulic acid, syringic acid, 3-hydroxycinnamic acid, and sinapic acid, indicating that the accumulation of these phenolic acids contributes to the reduction of oxidative damage induced by ROS under salt stress [31]. This study revealed the multi-dimensional regulatory network of Brassica napus in response to salt stress during germination and clarified the synergistic mechanism of metabolic reprogramming, gene expression regulation, and signal transduction. The core findings include: phenylpropanoid metabolism acts as a core defense hub, activates *PAL* (log_2_FC = 6.8) and *C4H* (log_2_FC = 2.5) through the ROS-ABA signaling cascade, drives lignin deposition (cinnamic acid accumulation 5.5 times) and antioxidant phenolic acid (such as ferulic acid + 11.4 times) synthesis, and alleviates ion toxicity [32]. Energy metabolism redirection is manifested as acute depletion of key intermediates in the TCA cycle (citric acid − 20.5 times, malic acid − 11.2 times), and activation of glycolysis (*PFK3* up-regulated 1.9 times) and proline synthesis (*P5CS* up-regulated 2.1 times) through SnRK1 kinase to balance osmotic regulation and energy supply needs [33]. The amino acid metabolic network showed asymmetric regulation: ornithine (+2,065,243-fold) enhanced membrane stability through polyamine synthesis, cysteine (+933,980-fold) as a glutathione precursor enhanced ROS scavenging capacity, and other amino acids were degraded through the GS/GOGAT cycle to maintain nitrogen redistribution [34].

A joint analysis of transcriptome and metabolome datasets indicated that “phenylpropanoid biosynthesis”, “amino acid biosynthesis”, and “carbon metabolism” are critical processes in the response of rapeseed (ZS11) seedlings to salt stress. The phenylpropanoid biosynthetic pathway is activated under abiotic stress conditions, including drought, heavy metals, salinity, extreme temperatures, and ultraviolet radiation. This activation leads to the accumulation of various phenolic compounds that may scavenge harmful reactive oxygen species [35]. Both the phenylpropanoid and flavonoid pathways play significant roles in mediating the response of common bean varieties to salt stress [36]. High expression of *AtRH17* confers salt stress tolerance through a novel response pathway involving nine genes, distinct from the ABA-dependent pathway. In this study, the expression of DIN2/BGLU30 and several other genes (*BnaC04G0279200ZS*, *BnaA09G0547400ZS*, *BnaA04G0017900ZS, BnaC08G0393700ZS*) was found to be up-regulated under salt stress conditions [37]. A comprehensive analysis of transcriptome and metabolome data revealed that amino acid metabolism is the most significant metabolic pathway in rapeseed roots under alkali-salt stress [28]. Comparative metabolic profiling indicated that amino acid metabolism and the TCA cycle are critical pathways in soybean’s response to salt stress [38]. Arabidopsis enhances its tolerance to salt stress mainly by regulating free proline accumulation and boosting ROS scavenging [39]. Under salt stress, the expression of proline synthesis-related genes (*BnaA05G0063500ZS*, *BnaC04G0569500ZS*, *BnaC04G0069800ZS*, *BnaA03G0194400ZS*, *BnaC03G0227700ZS*) was found to be up-regulated in rapeseed. The accumulation of carbohydrates plays a crucial role in helping plants regulate intracellular water potential and maintain stable cell expansion under salt stress. This mechanism also protects the integrity of membrane and protein structures from damage caused by osmotic regulation, thereby safeguarding normal cell function [40,41]. Under salt stress, Chinese cabbage exhibits an increased transcription level of genes associated with the photosynthetic apparatus and carbon metabolism, which may serve to mitigate damage to the photosynthetic system and enhance CO_2_ fixation and energy metabolism [42]. Additionally, moderate salt stress can stimulate the growth of Tartary buckwheat roots, elevate the levels of substances and enzyme activities related to carbon and nitrogen metabolism, and ultimately increase the yield of Tartary buckwheat per plant [43]. The new mechanism of salt stress adaptation in the germination stage of B.napus was revealed by multi-omics integration, and three breakthroughs were made compared with previous studies: (1) Development stage specificity: It was found that phenylpropanoid metabolism was dominant in the germination stage (cinnamic acid + 5.2 times), rather than the ion efflux mechanism of mature plants, and the TCA cycle had a threshold collapse under 200 mM NaCl (citric acid − 21.5 times) [44]; (2) Depth of multi-omics association: A total of 58 gene-metabolite functional pairs (such as PAL-cinnamic acid, r = 0.89) were identified, revealing the spatiotemporal coupling of metabolic reprogramming and transcriptional regulation, which is different from the ROS scavenging dominant strategy of cruciferous model plant Arabidopsis thaliana [45]; (3) Novel candidate gene mining: Through triple screening based on expression level (|log2FC| > 2), significance (FDR < 0.01), and correlation (|r| > 0.7), 44 key genes were pinpointed, including the phenylpropanoid regulatory gene *BnaC04G0279200ZS* (salt tolerance validated by homologous gene in rice) and the starch metabolism gene *BnaA09G0547400ZS* (not reported in previous omics data) [37].

This study identified 58 significantly related gene-metabolite pairs (|r| > 0.7, FDR < 0.05) by integrating transcriptome and metabolomics data. Among them, in the phenylpropanoid metabolic pathway, *BnaC04G0279200ZS* (*PAL*, log_2_FC = 7.9) was positively correlated with cinnamic acid (log_2_FC = 2.7) (r = 0.89), driving lignin deposition and antioxidant defense; in the proline metabolic pathway, *BnaA09G0547400ZS* (P5CS, log_2_FC = 7.7) was highly correlated with proline (log_2_FC = 1.7) (r = 0.85), maintaining osmotic regulation and active oxygen scavenging. A total of 78% of the association pairs were concentrated in phenylpropanoid, starch, and glutathione metabolic pathways. These associations reveal the synergistic mechanism of metabolic reprogramming and gene regulation. For example, the up-regulation of the *PAL* gene directly promotes cinnamic acid synthesis, while the activation of *P5CS* guarantees the continuous accumulation of proline [46]. In this study, functional coupling molecular modules were screened out through multi-omics integration, which provided a new strategy for screening salt-tolerant breeding targets. The relevant data are detailed in Tables S2 and S3. Through multi-omics analysis of the salt adaptation mechanism of *Brassica napus* at the germination stage, a multi-level breeding strategy was proposed: (1) CRISPR editing synergistically regulates *PAL* (log_2_FC = 6.8) and *NHX1* (log_2_FC = 2.6), drives lignin deposition (cinnamic acid + 5.5 times), knocks out *ProDH*, and overexpresses *P5CS* to construct a proline-ROS balance network [47]; (2) Using cinnamic acid and ornithine as metabolic markers, combined with machine learning models (such as citric acid-*IDH* association) to screen 300 + salt-tolerant germplasm [48]; (3) Cross-species validation of *PAL-NHX1* module, extended to rice and wheat [49]. By sharing multi-omics data and gene editing germplasm, integrating intelligent breeding technology, a synergistic paradigm of ‘metabolic homeostasis-dynamic activation‘ was formed to accelerate the field transformation of salt-tolerant varieties.

## 5. Conclusions

A total of 175 key DEMs involved in amino acid metabolism, carbohydrate metabolism, organic acid metabolism, phenolic acid metabolism, and the TCA cycle, as well as 44 key DEGs involved in hormone signal transduction, phenylpropanoid metabolism, carbohydrate metabolism, and amino acid metabolism were identified. The combined analysis of the transcriptome and metabolome revealed that rapeseed seedlings primarily engaged in phenylpropanoid metabolism, amino acid metabolism, and carbohydrate metabolism pathways in response to salt stress.

## Data Availability

The RNA-seq raw data for this study are stored in the National Genomics Data Center (https://bigd.big.ac.cn/gsa/browse/CRA020550, accessed on 20 November 2024) under accession number CRA020550. The metabolomics data can be found in the article and its Supplementary Materials.

## Supplementary Materials

The following supporting information can be downloaded at: https://www.mdpi.com/article/10.3390/cimb47070481/s1.
