# Supplementary material for: Metabolomics and Transcriptome Analysis of Rapeseed Under Salt Stress at Germination Stage
Source: Curr Issues Mol Biol. 2025 Jun 24;47(7):481. doi: 10.3390/cimb47070481 (PMC12293533; doi:10.3390/cimb47070481)
Supplement: Supplementary file 1 [file cimb-47-00481-s001.zip › Supplement Figures.pdf]

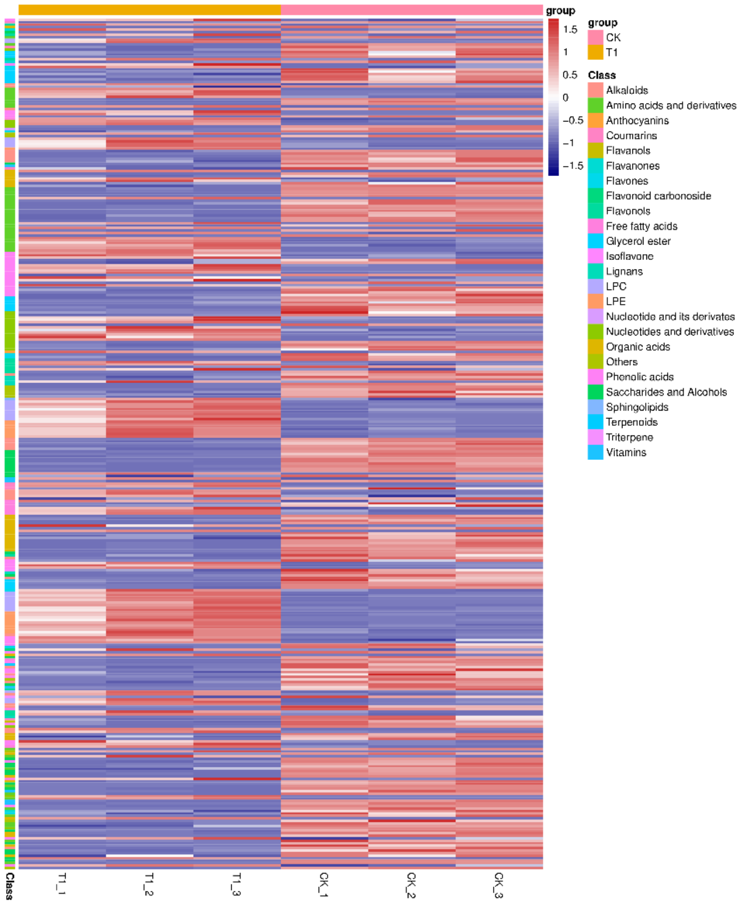


**Figure** S1. Cluster heat map of differential metabolites

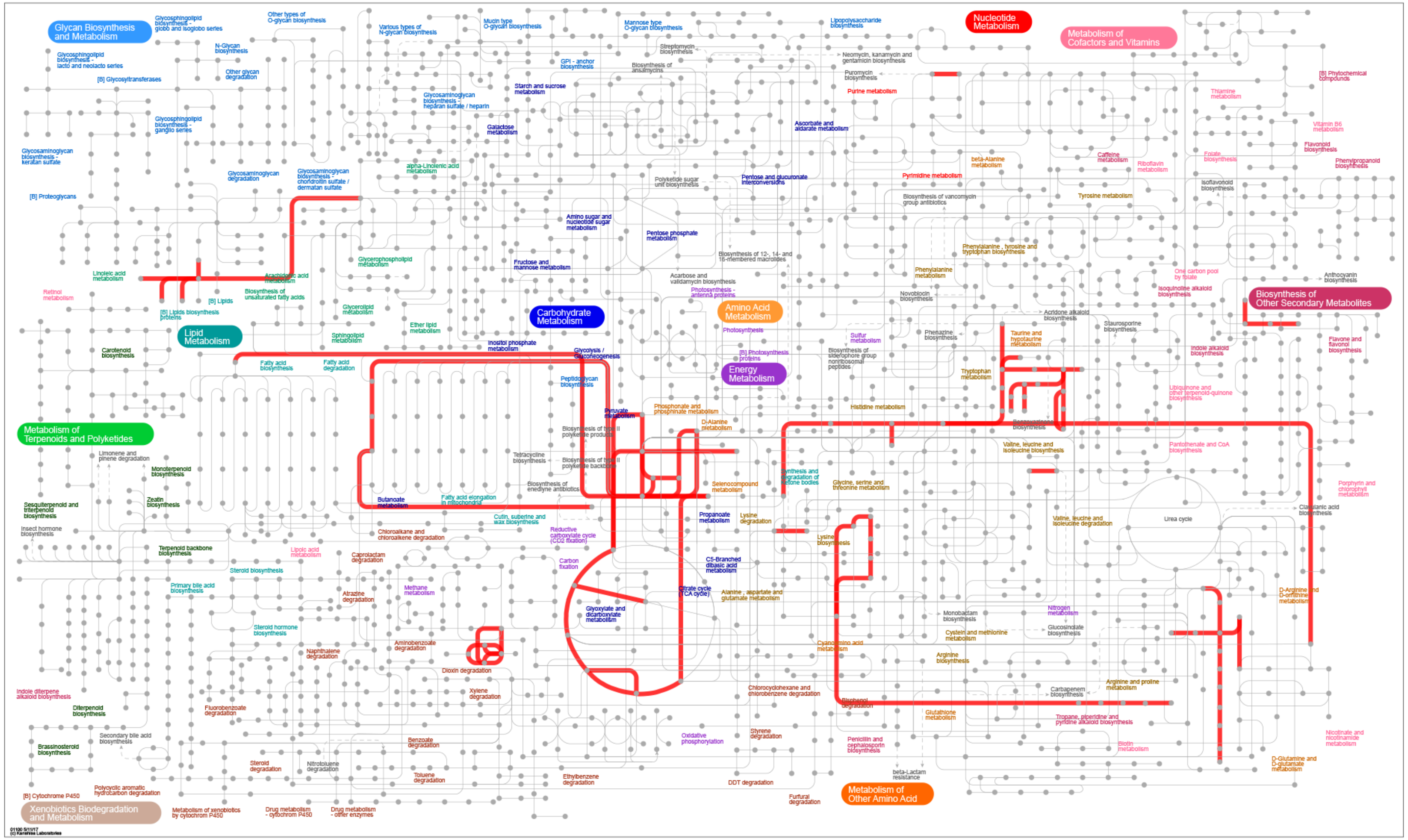


**Figure** S2. Metabolic pathway map

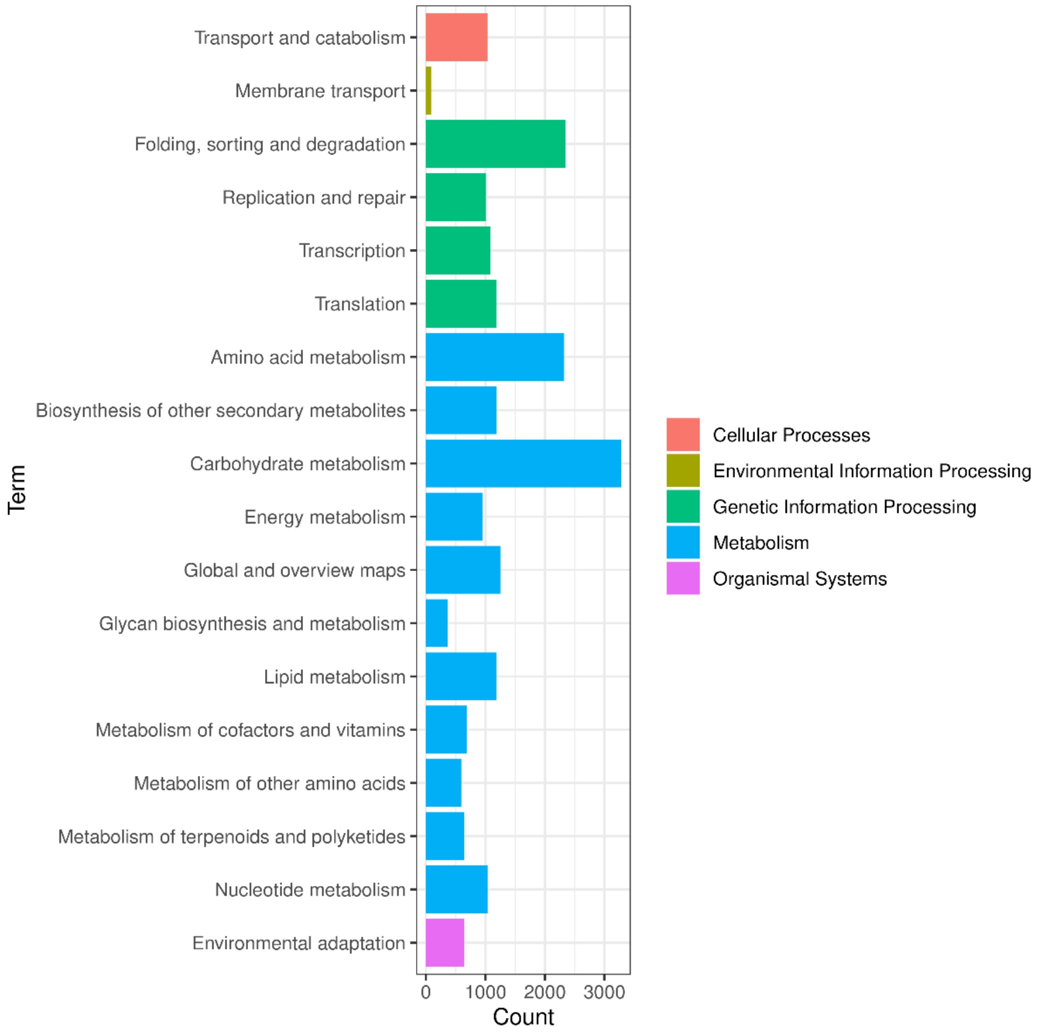


**Figure** S3. KEGG secondary classification map of genes

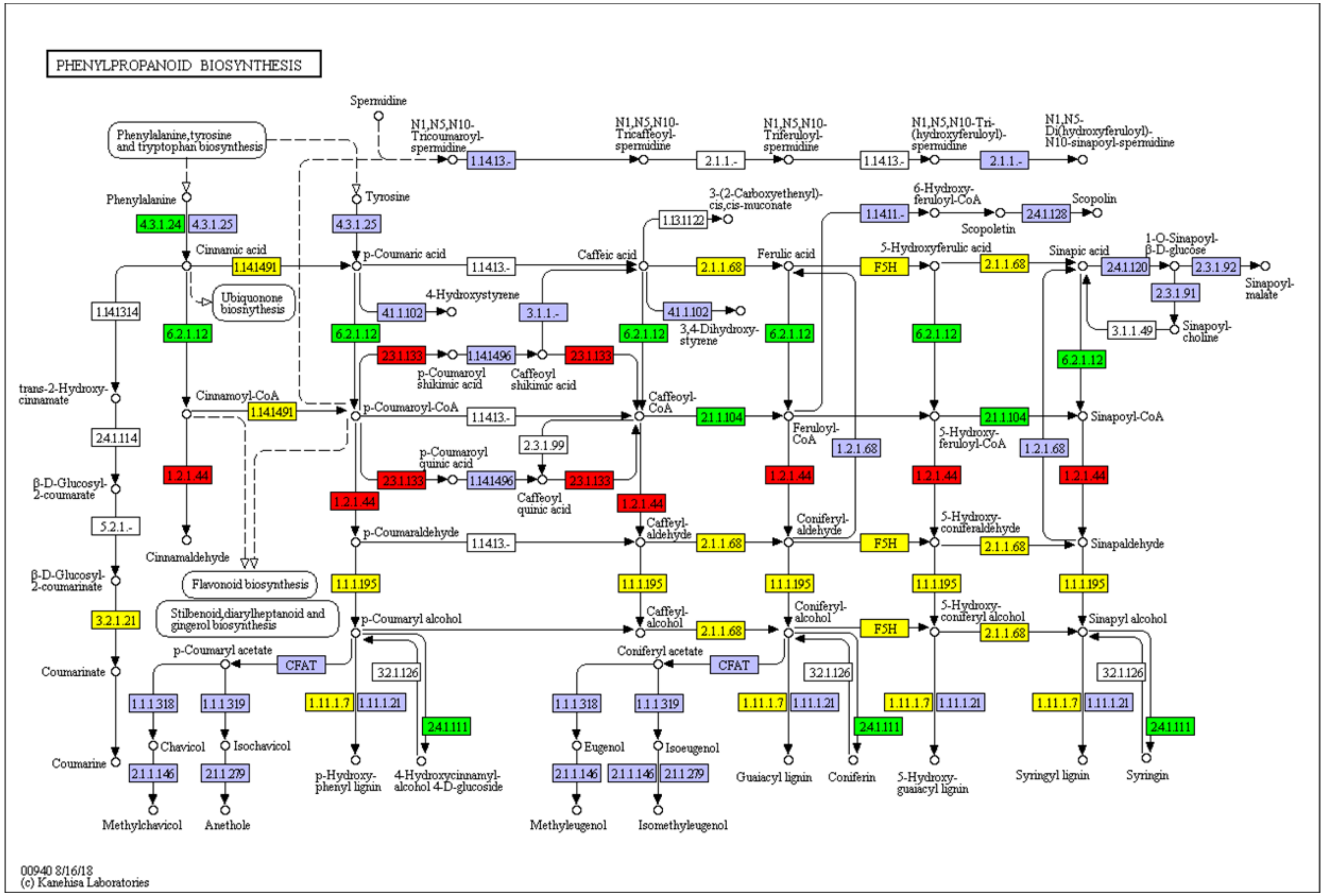


**Figure** S4. Gene phenylpropanoid biosynthesis pathway annotation map
